# Correlation between remnant thyroid gland I-131 uptake and serum thyroglobulin levels: can we rely on I-131 whole body scans?

**DOI:** 10.1186/s40644-024-00664-0

**Published:** 2024-01-30

**Authors:** Sang Hyun Hwang, KwanHyeong Jo, Jongtae Cha, Chun Goo Kang, Jiyoung Wang, Hojin Cho, Won Jun Kang, Arthur Cho

**Affiliations:** 1 Department of Nuclear Medicine, Severance Hospital, Yonsei University College of Medicine, 50-1 Yonsei-Ro, Seodaemun-Gu, Seoul, 03722 Republic of Korea; 2 Department of Nuclear Medicine, Korea University Guro Hospital, Korea University College of Medicine, Seoul, Republic of Korea; 3 Department of Nuclear Medicine, Severance Hospital, Seoul, Republic of Korea

**Keywords:** Radioiodine ablation, Papillary thyroid carcinoma, Thyroiditis, I-131 ablation

## Abstract

**Background:**

I-131 treatment (RAI) decision relies heavily on serum thyroglobulin (Tg) levels, as higher Tg levels are assumed to be correlated with higher I-131 uptake. Tg elevation, negative iodine scintigraphy (TENIS) definition is becoming more clinically relevant as alternative treatment methods are available. This study examined the correlation between Tg levels with I-131 uptake in remnant thyroid gland to evaluate the reliability of serum Tg levels in predicting I-131 uptake.

**Methods:**

From March 2012 to July 2019, 281 papillary thyroid cancer patients treated with 150 mCi RAI were retrospectively enrolled. Early (2nd day) and Delayed (7th day) post-RAI whole-body scan (WBS) neck counts were correlated with clinical and pathologic findings. Patients with normal neck ultrasound and undetectable level of serum Tg (< 0.2 ng/mL) and thyroglobulin antibody (TgAb) (< 10 IU/mL) were defined as ablation success within 2 years after I-131 ablation.

**Results:**

Thyroid gland weight, tumor size and thyroiditis were independent factors of preoperative serum Tg levels. Serum off-Tg levels correlated with Early and Delayed WBS neck counts, and thyroiditis pathology contributed to lower neck counts in both Early and Delayed WBSs. In multivariable analysis, Delayed WBS neck count, serum off-Tg and off-TgAb were significant factors for predicting ablation success.

**Conclusion:**

I-131 uptake and retention in remnant thyroid gland correlates with serum off-Tg levels, thyroiditis, and ablation success in thyroid cancer patients receiving high-dose I-131 therapy. Semi-quantitative I-131 analysis with Early and Delayed WBSs provides additional information in evaluating ablation success, with the potential application for metastasis treatment response evaluation.

**Supplementary Information:**

The online version contains supplementary material available at 10.1186/s40644-024-00664-0.

## Introduction

Post-operative surveillance for thyroid cancer is heavily dependent on serum thyroglobulin (Tg) levels. The current American Thyroid Association guidelines recommends periodical serum Tg measurements after total thyroidectomy along with thyroglobulin antibody (TgAb) and thyroid stimulating hormone (TSH) measurements combined with ultrasonogram for structural recurrence evaluation [1]. High-dose I-131 therapy (≥ 100 mCi) is recommended for high-risk patients, as it reduces recurrence rate and increases patient survival [2, 3] with the additional benefit of higher ablation success rates, which results in more reliable evaluation of serum Tg levels [4]. Excluding structural recurrence, clinicians depend mostly on rising serum Tg levels to determine repeat radioactive iodine (RAI) therapy. However, there are limitations of relying only on serum Tg levels, as the reliability of Tg measurements in patients with elevated TgAb is reduced, and also considering that some patients are Tg seronegative despite I-131 positive whole body scans (WBS). Therefore, the evaluation of clinical factors influencing I-131 uptake and serum Tg levels may be helpful, especially as lenvatinib is indicated for RAI-refractory (RAI-R) thyroid metastasis, of which I-131 uptake and serum Tg levels has not been clearly defined [5, 6].

A clinical unmet need in repeated I-131 treatment decision is the discordance between I-131 uptake on WBS and serum Tg levels in the absence of structural evidence of metastasis. Major factors contributing to this discordance are biologic factors, such as loss of iodine organification ability, de-differentiation resulting in reduced sodium iodide symporter (NIS) expression, and physical limitations of low resolution of I-131 WBS. Without biopsy of each metastatic site, it is difficult to determine which factors contribute to the discordance between I-131 uptake on WBS and serum Tg levels in metastatic lesions. However, it is possible to evaluate the concordance between I-131 uptake with serum Tg levels by evaluating I-131 uptake in the remnant thyroid gland during remnant thyroid gland ablation. This will overcome the previously mentioned limitations in metastasis evaluation, as the remnant thyroid gland retains original thyroid gland functionality and I-131 soft tissue attenuation is minimal because of similar depth of the remnant thyroid gland. An analysis on serum Tg levels, I-131 uptake in functioning remnant thyroid gland, and pathologic specimen findings may provide additional information on the reliability of I-131 uptake on WBS with serum Tg levels.

This study aimed to correlate I-131 uptake in the remnant thyroid gland with TSH-stimulated Tg levels (off-Tg) and evaluate available clinical and pathologic findings that may influence RAI ablation success in a fully functional thyroid gland in real-world clinical setting, which may be helpful in determining repeat I-131 treatment in thyroid cancer RAI ablation or metastasis.

## Materials and methods

### Patient selection and follow-up

Patient selection was targeted at controlling factors that influence I-131 uptake and retention. We selected only patients receiving high-dose I-131 ablation (150 mCi) to control for administrated dose and thyroid uptake. We also selected patients who received post-ablation I-131 WBS on the 2nd day and 7th day post-administration to control for changes in biodistribution on different days of WBS scan during semi-quantitative evaluation of I-131 retention. To fully correlate serum Tg levels with I-131 uptake in the remnant thyroid gland, we excluded all patients who had metastasis during I-131 WBS or newly developed metastasis during the follow-up period. Additionally, we included patients who had pre-operative serum Tg levels, thyroid weight, and tumor size on pathologic reports, in order to evaluate whether there is a correlation between serum Tg levels and thyroid gland size.

From March 2012 to July 2019, a total of 1,319 patients were admitted to our hospital for high-dose (150 mCi) I-131 ablation. Of these patients, 1,066 patients had WBS on the 7th day, and 706 of these patients had WBS on the 2nd day. Of these 706 patients, 47 patients were excluded due to administration of recombinant human TSH, 80 patients were excluded as these patients received prior I-131 therapy, 89 patients were excluded for having > 150 mCi I-131 re-treatment for metastasis, and 130 patients were excluded due to insufficient data for follow-up (either less than 6 months of follow-up or fewer than two times of follow-up measurements). Finally, 79 patients were excluded for insufficient pre-operative data (nine patients for no preoperative serum Tg, 66 patients for no thyroid gland weight on pathologic report, and four patients for no iodine/creatinine ratio). Finally, this study enrolled 281 patients who had no known metastasis, received first I-131 therapy, and had follow-up serum TSH, Tg, and TgAb level detected for at least 2 years at approximately 6-month interval as routine clinical follow-up protocol. The patients’ TNM stage, serum TSH, Tg, and TgAb levels acquired on ablation day, and lymphocytic thyroiditis determined on pathologic slides were recorded. Successful thyroid remnant ablation (golden standard) was defined as no structural lesion on neck ultrasound and undetectable serum Tg levels during levothyroxine administration (on-Tg) (< 0.2 ng/mL) and TgAb (< 10 IU/mL) between 6 months and 2 years after treatment. The institutional review board of our university approved this retrospective study, and the requirement to obtain informed consent was waived (IRB approved no. 4–2021-0257).

### Radioactive-iodine remnant ablation protocol

Before RAI remnant ablation therapy, all patients discontinued levothyroxine supplement for 5 weeks with triiodothyronine replacement for the initial 3 weeks. Patients underwent strict iodine restriction diet for 2 weeks before I-131 administration, which ended 3 days after RAI administration. TSH levels were confirmed to be at least 30 mIU/L before admission. All patients underwent two post-RAI WBSs after hospital discharge. The first WBS was performed on the day of discharge (2nd day after I-131 administration; Early scan) and on the 7th day after I-131 administration (Delayed scan).

### Post-RAI whole body scan analysis

Early and Delayed post-RAI WBSs were acquired using a gamma camera equipped with a high-energy parallel hole collimator (Infinia, GE Medical Systems, Milwaukee, WI, USA). Early and Delayed scans obtained from a total of 281 enrolled patients were analyzed. The count of these two scans was measured by Xeleris workstation (GE Medical Systems) using the following method: A circular region of interest (ROI) was drawn on the neck on the Early scan to encompass the remnant thyroid, and the same size ROI was copied and pasted onto the neck of the Delayed scan. The same sized ROI was used for all patients, and the total counts in each ROI was recorded. Also, since the whole body counts were higher at Early scan compared to Delayed scan, Early WBS scan was acquired faster than Delayed scan. To compare the thyroid uptake in Early scan compared to Delayed scan, both Early scan speed and Delayed scan speed were factored into analysis. This was done by first recording the scan speed on DICOM header (Tag (0008,0008), which records scan speed as mm/sec), and then dividing the neck ROI counts by scan speed for both Early and Delayed scans. Finally, Early scan counts were decay corrected to Delayed scan. This was done using the following formula:

$$\begin{array}{c}\mathrm{Decay \,corrected \,Activity }\left({\text{counts}}\right)=Ao\left(Early \,scan \,counts\right)\times {e}^{-\left(\frac{0.693}{8.04}\right)\times Time }\\ \mathrm{Time }= (\mathrm{day \,interval \,between \,Early \,and \,Delayed \,scan})\end{array}$$

Finally, the reduction ratio was calculated using the following formula: (Delayed WBS neck count—Early WBS neck count)/Early WBS neck count * 100.

### Statistical analysis

Pearson's chi-square test or linear-by-linear association test was used to compare differences in qualitative variables, and Mann–Whitney U test or Kruskal–Wallis test was used to compare differences in continuous variables. Simple and multiple regression analyses were performed to evaluate the factors influencing preoperative serum Tg levels. Serum off-Tg level were categorized with quartile, and Early and Delayed WBS neck counts of each group were compared by Kruskal–Wallis test and Dunn’s post-hoc analysis. Early and Delayed WBS neck counts were divided into quartiles and evaluated. Univariable and multivariable logistic regression analyses were performed to evaluate the prediction of ablation success. *P* values less than 0.05 were on univariable analysis were included in the multivariable analysis. With respect to post-RAI WBS parameters, receiver-operating characteristic (ROC) curve using Youden index was performed to determine the cut-off values for predicting ablation success. DeLong test was used to compare the difference between the area under the ROC (AUROC) curve of different models. Factors significantly associated with ablation success in the univariable analysis were selected for constructing a nomogram. The Hosmer–Lemeshow test and calibration curve were employed to assess the model calibration. Internal validation was performed using 1,000 bootstrap resampling. All statistical analyses were conducted using R version 4.0.4 (R Foundation for Statistical Computing, Vienna, Austria), and GraphPad Prism version 9.0 (GraphPad Software, San Diego, CA, USA) was used for figures. *P* values less than 0.05 were considered statistically significant.

## Results

### Patient characteristics

The characteristics of the 281 patients enrolled in this study are shown in Table 1. The median age at the time of RAI remnant ablation was 38 years (range 16–75), and 196 patients (69.8%) were female. The median weight of the resected thyroid gland was 19.0 g (range 4.2–127.0), and the median tumor size was 1.3 cm (range 0.3–7.0). In post-RAI WBS analysis, the median Early and Delayed neck counts were 49,823 range (656–560,227) and 2,379 (85–46,212), respectively. The median reduction ratio was 95.2% (range 70.1–99.3). Seventy patients (24.9%) had lymphocytic thyroiditis on pathologic report, and 157 patients (55.9%) had ablation success.

**Table 1:** Patient demographics of the 281 enrolled patients

Characteristics	Number of patients (%)
Age (years)	38 (16–75)
Sex	
Male:Female	85 (30.2%):196 (69.8%)
Thyroid gland weight (g)	19.0 (4.2–127.0)
Primary tumor size (cm)	1.3 (0.3–7.0)
Pathologic TNM stage	
T1aN1b	25 (8.9%)
T1bN1b	10 (3.6%)
T2N1b	5 (1.8%)
T3N0	2 (0.7%)
T3N1a	8 (2.8%)
T3N1b	231 (82.2%)
ATA risk stratification	
Intermediate risk	196 (69.8%)
High risk	85 (30.2%)
Assay at ablation	
TSH (mIU/mL)	51.31 (30.13–158.81)
Tg (ng/mL)	2.5 (0.039–280.0)
TgAb (IU/mL)	10.9 (9.9–1,859.0)
I-131 WBS analysis	
Early WBS neck count^a^	49,823 (656–560,227)
Delayed WBS neck count^a^	2,379 (85–46,212)
Reduction ratio (%)	95.2 (70.1–99.3)
Interval between surgery and ablation (months)	3.1 (1.5–8.5)
Thyroiditis (Present:None)	70 (24.9%):211 (75.1%)
Ablation success: Ablation failure	157 (55.9%):124 (44.1%)

Continuous values are presented as median (range)

*ATA* American Thyroid Association, *TSH* thyroglobulin stimulating hormone, *Tg* thyroglobulin, *TgAb* thyroglobulin antibody, *WBS* whole body scan

^a^Scan speed adjusted neck count = neck counts/scan speed (mm/sec)

### Factors associated with preoperative serum Tg levels

An initial assessment of known clinicopathological factors contributing to pre-operative serum Tg levels was assessed to evaluate for the correlation between serum Tg levels with thyroid gland size. Univariate analysis was performed to assess the association between preoperative serum Tg levels and clinical and pathologic factors. Thyroid gland weight (β = 3.68, *p* < 0.001), thyroiditis (β = -29.51, *p* = 0.027), and tumor size (β = 41.7, *p* < 0.001) were significantly correlated with preoperative serum Tg levels, while TNM stage was not (Supplementary Table 1). Multivariable analysis was performed using other statistically significant factors in univariable analysis, and thyroid gland weight, tumor size and thyroiditis were independent determinants of preoperative serum Tg levels (β = 2.47, β = 27.0, β = -25.83, respectively).

### Serum off-Tg level and WBS neck count

**Fig. 1:**
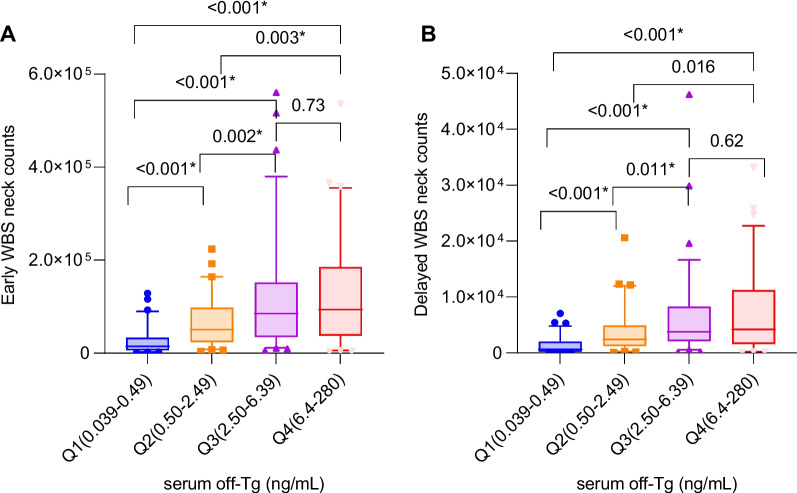
Box plot of Early whole body scan (WBS) neck count (**A**) and Delayed WBS neck count (**B**) according to serum off-thyroglobulin (Tg) levels. Serum off-Tg levels were divided into quartiles

When serum off-Tg levels were grouped into quartiles (Q1 (0.039–0.49 mg/mL), Q2 (0.50–2.49 mg/mL), Q3 (2.50–6.39 mg/mL), and Q4 (6.40–280.0 mg/mL)), there was a statistically significant increase in neck uptake as serum off-Tg levels increased (Fig. 1). This trend was seen in both Early and Delayed WBS, except between Q3 and Q4, which confirms there is a correlation between functional remnant thyroid gland and serum Tg levels. Early WBS neck counts in each serum off-Tg quartile groups were 14,614 counts/mm/sec (interquartile range [IQR] 6,572–33,011) (Q1), 50,647 counts/mm/sec (IQR 24,377–97,466) (Q2), 85,090 counts/mm/sec (IQR 35,594–151,606) (Q3), and 93,690 counts/mm/sec (IQR 38,964–185,066) (Q4). The Delayed WBS neck counts of quartiles of serum off-Tg were 653 counts/mm/sec (IQR 248–1,970) (Q1), 2,409 counts/mm/sec (IQR 1,264–4,875), 3,776 counts/mm/sec (IQR 2,092–8,131) (Q3), and 4,175 counts/mm/sec (1,587–10,608) (Q4). In Early WBS neck count, Q1 was significantly lower than Q2, Q3, and Q4 in post-hoc analysis (*p* < 0.001, respectively), and Q2 was significantly lower than Q3, and Q4 (*p* = 0.002, *p* = 0.003, each). In Delayed WBS neck count, Q1 was significantly lower than Q2, Q3, and Q4 (*p* < 0.001, respectively), and Q2 was significantly lower than Q3 (*p* = 0.011).

### Correlation between WBS neck count and pathologic and clinical parameters

Further analysis was performed to evaluate for known clinicopathologic factors contributing to I-131 uptake and serum off-Tg levels (Supplementary Table 2). When Early WBS neck counts were grouped into quartiles, there was a significant trend of increase in the percentage of thyroiditis patients with lower neck counts and a lower percentage of thyroiditis patients in patients with high neck counts (Q1 49.3% (35/71), Q2 31.4% (22/70), Q3 15.7% (11/70), and Q4 2.9% (2/70), *p* < 0.001, by linear-by-linear association). Additionally, there was a decreasing trend in female proportion as early WBS neck counts increased (*p* < 0.001). There was also a positive correlation between serum off-Tg levels and Early WBS neck counts, which also contributes to the assumption that I-131 WBS correlates with functional remnant thyroid gland.

Delayed WBS neck counts also showed similar inverse correlation between thyroiditis percentage, proportion of female population, and neck counts, as well as a positive correlation between off-Tg levels and neck counts (Supplementary Table 2).

**Fig. 2:**
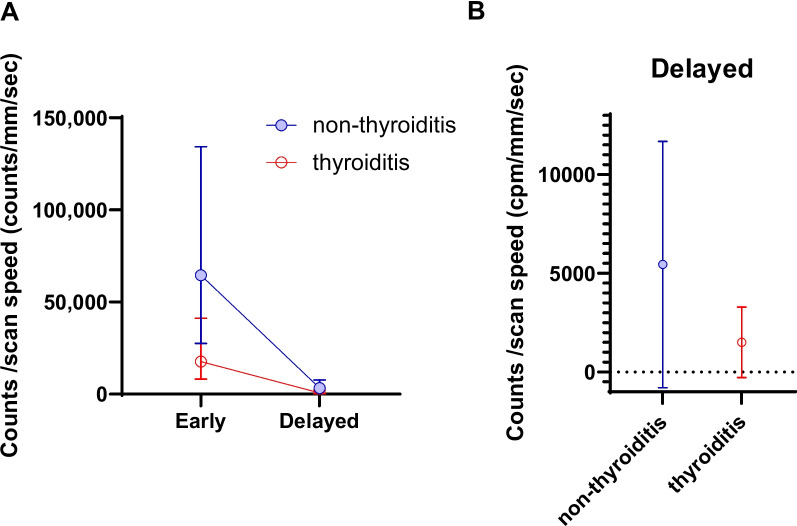
Early and Delayed whole body scan (WBS) neck counts in patients with and without thyroiditis (**A**). Lower I-131 uptake was observed in thyroiditis patients compared to non-thyroiditis patients in both Early and Delayed WBS (**B**)

There was no difference in iodine/creatinine ratio between each quartile group of both Early and Delayed WBS neck counts. We re-organized the data to evaluate I-131 uptake according to thyroiditis. Thyroiditis patients had significant lower neck counts in both Early (64,554 vs. 17,741 counts/mm/sec, *p* < 0.001) and Delayed (3,279 vs. 738 counts/mm/sec, *p* < 0.001) scans. However, the reduction ratio of each group (95.2% vs. 95.4%) was not statistically significant (*p* = 0.422) (Fig. 2).

### Prediction of ablation success

ROC curve analysis was performed to evaluate for the optimal cut-off values for serum Tg level (4.7 ng/mL), TgAb level (19.2 IU/mL), Early WBS neck count (51,814 counts/mm/sec), Delayed neck count (4,376 counts/mm/sec), and reduction ratio (-95.8%). Using these values, univariable logistic regression analysis showed that coexisting thyroiditis, serum Tg, serum TgAb, Early WBS neck count, Delayed WBS neck count, and reduction ratio were significant factors for ablation success (*p* < 0.05, respectively, Table 2, Supplementary Fig. 1).

**Table 2:** Univariable and multivariable analyses in predicting ablation success

Variable	Univariable analysis		Multivariable analysis							
			Model 1		Model 2		Model 3		Model 4	
	OR (95% CI)	*P* value	OR (95% CI)	*P* value	OR (95% CI)	*P* value	OR (95% CI)	*P* value	OR (95% CI)	*P* value
Age	1.02 (1.00–1.04)	0.100								
Sex (vs. male)	0.97 (0.58–1.61)	0.894								
Pathology										
Primary tumor size (cm)	0.85 (0.66–1.08)	0.189								
T3N0-1b (vs. T1a-2N1b)	0.93 (0.46–1.81)	0.823								
Thyroiditis present (vs. absent)	0.58 (0.33–0.99)	**0.049**	0.77 (0.34–1.83)	0.545	0.93 (0.4–2.2)	0.88	0.98 (0.42–2.3)	0.96	0.93 (0.40–2.31)	0.878
Assay at ablation										
Tg (vs. > 4.7 ng/mL)	7.31 (4.20–13.10)	** < 0.001**	12.26 (6.49–24.14)	** < 0.001**	14.72 (7.31–29.62)	** < 0.001**	17.59 (8.28–37.37)	** < 0.001**	18.07 (8.77–40.45)	** < 0.001**
TgAb (vs. > 19.2 IU/mL)	5.58 (2.99–10.94)	** < 0.001**	8.99 (3.90–22.36)	** < 0.001**	8.12 (3.36–19.62)	** < 0.001**	7.91 (3.25–19.23)	** < 0.001**	7.13 (2.99–18.30)	** < 0.001**
I-131 WBS analysis										
Early WBS neck count^a^ (vs. ≤ 51,814)	1.75 (1.09–2.82)	**0.022**			2.16 (1.12–4.2)	**0.022**			1.14 (0.48–2.70)	0.772
Delayed WBS neck count^a^ (vs. ≤ 4,376)	2.24 (1.46–3.84)	**0.003**					3.59 (1.67–7.74)	** < 0.001**	2.93 (1.11–8.05)	**0.032**
Reduction ratio (vs. > 95.8) (%)	1.75 (1.07–2.87)	**0.027**							1.61 (0.85–3.07)	0.143
Interval between surgery and ablation (months)	0.98 (0.79–1.24)	0.887								
AUROC curve (95% CI)			0.803 (0.752–0.855)		0.828 (0.779–0.876)		0.842 (0.796–0.888)		0.844 (0.798–0.891)	

*OR* odds ratio, *CI* confidence interval, *Tg* thyroglobulin, *TgAb* thyroglobulin antibody, *WBS* whole body scan, *AUROC* area under the receiver operating characteristic

^a^Scan speed adjusted neck count = neck counts/scan speed (mm/sec)

Based on the univariable analysis results, four different multivariable models were established to compare the additive predictive power of WBS parameters. Model 1 showed that the known factors for ablation success, serum Tg (odds ratio [OR] = 12.26, 95% confidence interval [CI] = 6.49–24.14, *p* < 0.001), and serum TgAb (OR = 8.99, 95% CI = 3.90–22.36, *p* < 0.001) were significant factors for predicting ablation success. Model 2 added Early WBS neck counts to Model 1 factors, and showed that serum Tg (OR = 14.72, 95% CI = 7.31–29.62, *p* < 0.001), serum TgAb (OR = 8.12, 95% CI = 3.36–19.62, *p* < 0.001), and Early neck counts (OR = 2.16, 95% CI = 1.12–4.2, *p* = 0.022) were significant factors for predicting ablation success. Model 3 added Delayed WBS neck counts to Model 1 factors, and showed that serum Tg (OR = 17.59, 95% CI = 8.28–37.37, *p* < 0.001), serum TgAb (OR = 7.91, 95% CI = 3.25–19.23, *p* < 0.001), and Delayed neck counts (OR = 3.59, 95% CI = 1.67–7.74, *p* = 0.011) were significant factors for predicting ablation success. Model 4 included all WBS parameters, and showed that serum Tg (OR = 18.07, 95% CI = 8.77–40.45, *p* < 0.001), serum TgAb (OR = 7.13, 95% CI = 2.99–13.30, *p* < 0.001), and Delayed neck counts (OR = 2.93, 95% CI = 1.11–8.05, *p* = 0.032) were significant factors for predicting ablation success. The AUROC curve of each model was 0.803 (95% CI = 0.752–0.855 for Model 1), 0.828 (95% CI = 0.779–0.876 for Model 2), 0.842 (95% CI = 0.796–0.888 for Model 3) and 0.844 (95% CI = 0.798–0.891 for Model 4). The DeLong test showed that models with delayed or all WBS parameters (Models 3,4) were more effective in predicting ablation success (*p* = 0.003, 0.006, respectively) compared to those without WBS parameters (Model 1) in predicting ablation success. Although Early WBS neck count was significantly correlated with ablation success in multivariable analysis (Model 2), there was no significant difference in predicting ablation success compared to Model 1 (Delong test *p* = 0.09).

**Fig. 3:**
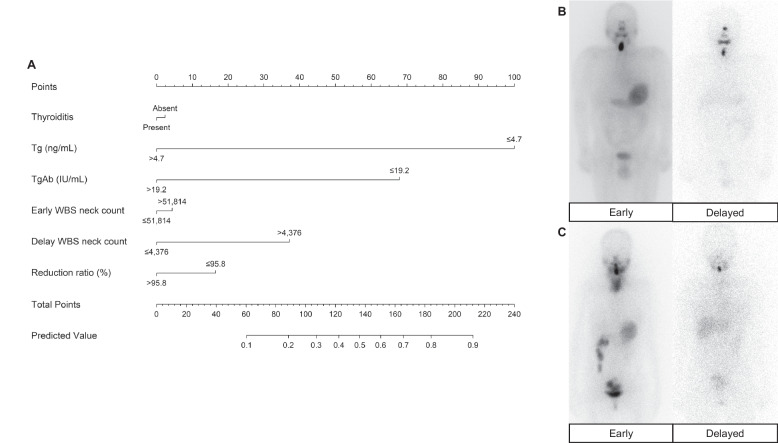
Nomogram to predict ablation success for patients with thyroid cancer receiving high-dose I-131 therapy based on multivariable analysis and example cases. **A** Nomogram. Each factor was translated into points by drawing a line straight up, after which all points were added to a total point score. The total score was then translated into the predictive value axis by drawing a line straight down to estimate the ablation success probability. **B** A 64-year-old male patient with TSH, Tg and TgAb levels of 40.1 mIU/L, 0.1 ng/mL and 28.14 IU/mL at ablation and no evidence of thyroiditis on pathology. Neck counts were 128,809 on Early scan and 5,438 on Delayed scan, with 95.8% reduction rate. This patient had ablation success. Nomogram total points were 2.5 + 100 + 0 + 5 + 37.5 + 17.5 = 162.5, which translates into a predictive value of 68% of ablation success. **C** A 40-year-old female patient without thyroiditis on pathology. During ablation, TSH, Tg and TgAb levels were 39.0 mIU/L, 5.1 ng/mL and 9.9 IU/mL, and neck counts were 51,791 on Early scan and 2,406 on Delayed scan, with 95.4% reduction rate. This patient had ablation failure. Nomogram total points were 0 + 0 + 67.5 + 0 + 0 + 17.5 = 85, which translates into a predictive value of 18% of ablation success

Based on these results, the variables significantly associated with ablation success in the univariable analysis (Model 4) were used in the construction of the nomogram (Fig. 3). The Hosmer − Lemeshow test demonstrated that the model was a good fit (*p* = 0.961). Moreover, calibration plots for bootstrap resampling validation demonstrated good consistency (Supplementary Fig. 2).

## Discussion

The present study evaluated the clinical relevance of semi-quantitative evaluation of I-131 uptake in the remnant thyroid using routine Early and Delayed I-131 WBS acquired in the clinical setting, which may be helpful in providing baseline information for evaluating I-131 uptake and retention in the thyroid. To semi-quantitatively evaluate I-131 uptake and retention, we controlled for administrated dose and biodistribution changes by selecting patients receiving 150 mCi and I-131 WBS acquired on the same days, and demonstrated that the thyroid uptake in Delayed I-131 WBS contributed to predicting ablation success. To our knowledge, this study is the first to evaluate the correlation between quantified I-131 neck uptake and thyroiditis pathology with ablation success in patients with remnant thyroid gland only. Considering the different contribution of I-131 neck counts in early and delayed scans in ablation success evaluation, dual timepoint I-131 imaging, especially dual timepoint single-photon emission computed tomography/computed tomography (SPECT/CT), may also be useful in metastasis treatment response evaluation and in the characterization of neck nodules found in post-operative neck ultrasonogram. I-131 SPECT/CT will be needed for semi-quantification analysis of individual metastatic lesions, as well as to account for different soft tissue attenuation correction. Potentially, dual time point SPECT/CT analysis may show different retention patterns of each lesion, which may be correlated with iodine organification ability, and may better predict therapy response.

To confirm that serum Tg levels reflect thyroid function, we first evaluated the factors contributing to serum Tg levels in the preoperative thyroid glands. In line with previous reports, we have shown that preoperative serum Tg levels are positively correlated with thyroid gland size and tumor burden, and inversely correlated with thyroiditis [7–9]. After confirming that serum Tg levels correlate with thyroid gland size in our patient population, we then proceeded to evaluate the factors correlating with I-131 uptake in the remnant thyroid gland. We found a general trend of rising serum off-Tg levels in both Early and Delayed WBS counts, confirming previous reports that I-131 uptake reflects remnant thyroid gland amount [10–12]. In regard to ablation success, our results support previous studies showing that serum off-Tg levels are predictive of ablation success [9, 13].

A major finding in the semi-quantification analysis of I-131 uptake in the remnant gland was that compared to Early scans, a very small remnant amount of I-131 uptake was retained in Delayed scans, as less than 5% of I-131 uptake was retained in Delayed scans. Multivariable analysis revealed that I-131 retention contributed more to ablation success in Delayed scans compared to Early scans. We speculate that the I-131 retention capability of the thyroid gland contributes more than initial Early scans in thyroid ablation. Our findings could potentially be applied to metastatic lesions treatment response evaluation, as metastasis “retaining” I-131 seen on Delayed scans may be a more important factor than in Early scans in treatment response prediction. A potential application of our study could be that semi-quantitative analysis using Early and Delayed I-131 or I-123 SPECT/CT scans might predict therapy response or RAI-R.

Another major finding with I-131 semi-quantification is that patients with thyroiditis on pathology had very low iodine concentration ability, as there was a significant difference in I-131 counts in both Early and Delayed scans. Our results confirm previous studies that showed low ablation success rates in patients with Hashimoto’s thyroiditis (HT) [11, 14], and also provide supporting clinical data of reduced iodine concentration ability of HT [15]. However, complete ablation may have minimal clinical significance, as HT is a favorable prognostic factor for thyroid cancer prognosis [16].

The present study had several limitations. First, this was a retrospective, single center study with a relatively short interval follow-up period. Second, we provided a rather strict definition of ablation success, as we defined undetectable TgAb levels for ablation success. This was to ensure that serum Tg levels were not influenced by serum TgAb levels, and to correlate the neck counts more precisely with Tg levels. This may contribute to the lower ablation success rates compared to previous studies, but may more accurately evaluate TgAb level disappearance after I-131 administration. Third, we were not able to use I-131 standard during I-131 WBS for absolute I-131 quantification in routine clinical setting; however, we partially corrected for this by adjusting for scan time and scanner speed.

## Conclusion

The I-131 uptake and retention in the remnant thyroid gland correlates with the serum off-Tg levels, thyroiditis, and ablation success in thyroid cancer patients receiving high-dose I-131 therapy. Semi-quantitative I-131 analysis with Early and Delayed WBSs may provide additional information in evaluating ablation success, with the potential application for metastasis treatment response evaluation.

### Supplementary Information

**Additional file 1: Supplementary Figure 1.** Representative cases of thyroid remnant retention in I-131 whole body scan (WBS) and spot views in patients with and without thyroiditis. **Supplementary Figure 2.** Calibration plots of the nomogram. **Supplementary Table 1.** Analysis of preoperative serum Tg level associated with pathologic and clinical parameters. **Supplementary Table 2.** Correlation between whole body scan neck count and pathologic and clinical parameters.

## Data Availability

The datasets used and/or analyzed during the current study are available from the corresponding author upon reasonable request.

## Abbreviations

RAI: Radioactive iodine
RAI-R: Radioactive iodine-refractory
Tg: Thyroglobulin
TgAb: Thyroglobulin antibody
TSH: Thyroid stimulating hormone
NIS: Sodium iodide symporter
WBS: Whole-body scan
OR: Odds ratio
CI: Confidence interval
IQR: Interquartile range
TENIS: Tg elevation, negative iodine scintigraphy
ROC: Receiver-operating characteristic
SPECT/CT: Single-photon emission computed tomography/computed tomography
HT: Hashimoto’s thyroiditis
