# Supplementary material for: Correlation between remnant thyroid gland I-131 uptake and serum thyroglobulin levels: can we rely on I-131 whole body scans?
Source: Cancer Imaging. 2024 Jan 30;24:21. doi: 10.1186/s40644-024-00664-0 (PMC10826009; doi:10.1186/s40644-024-00664-0)
Supplement: Supplementary file 1 — Additional file 1: Supplementary Figure 1. Representative cases of thyroid remnant retention in I-131 whole body scan (WBS) and spot views in patients with and without thyroiditis. Supplementary Figure 2. Calibration plots of the nomogram. Supplementary Table 1. Analysis of preoperative serum Tg level associated with pathologic and clinical parameters. Supplementary Table 2. Correlation between whole body scan neck count and pathologic and clinical parameters. [file 40644_2024_664_MOESM1_ESM.docx]

**Supplementary Figure 1.** Representative cases of thyroid remnant retention in I-131 whole body scan (WBS) and spot views in patients with and without thyroiditis. All spot views were acquired until 300,000 counts were detected. No evidence of recurrence was seen on follow-up ultrasonogram.

(A-B) A 26-y-old woman with papillary thyroid carcinoma had bilateral total thyroidectomy with central compartment neck dissection and right modified radical neck dissection. This patient had no evidence of thyroiditis. Early WBS (A) neck count was 81,565 (counts/mm/sec) and Delayed WBS (B) neck count was 6,395 (counts/mm/sec). Reduction ratio was 92.2%. After radioactive iodine (RAI) ablation, serum Tg and TgAb level were <0.1 ng/mL and <10 IU/mL, respectively.

(C-D) A 47-y-old woman with papillary thyroid carcinoma had bilateral total thyroidectomy with central compartment neck dissection and left modified radical neck dissection. This patient had pathologic proven thyroiditis. Early WBS (C) neck count was 18,885 (counts/mm/sec) and Delayed delayed WBS (D) neck count was 573 (counts/mm/sec). Reduction ratio was 95.9%. After RAI ablation, follow-up serum Tg and TgAb level were 1.2 ng/mL and 10.5 IU/mL, respectively. Notice the low I-131 uptake in the whole body compared to non-thyroiditis patients.


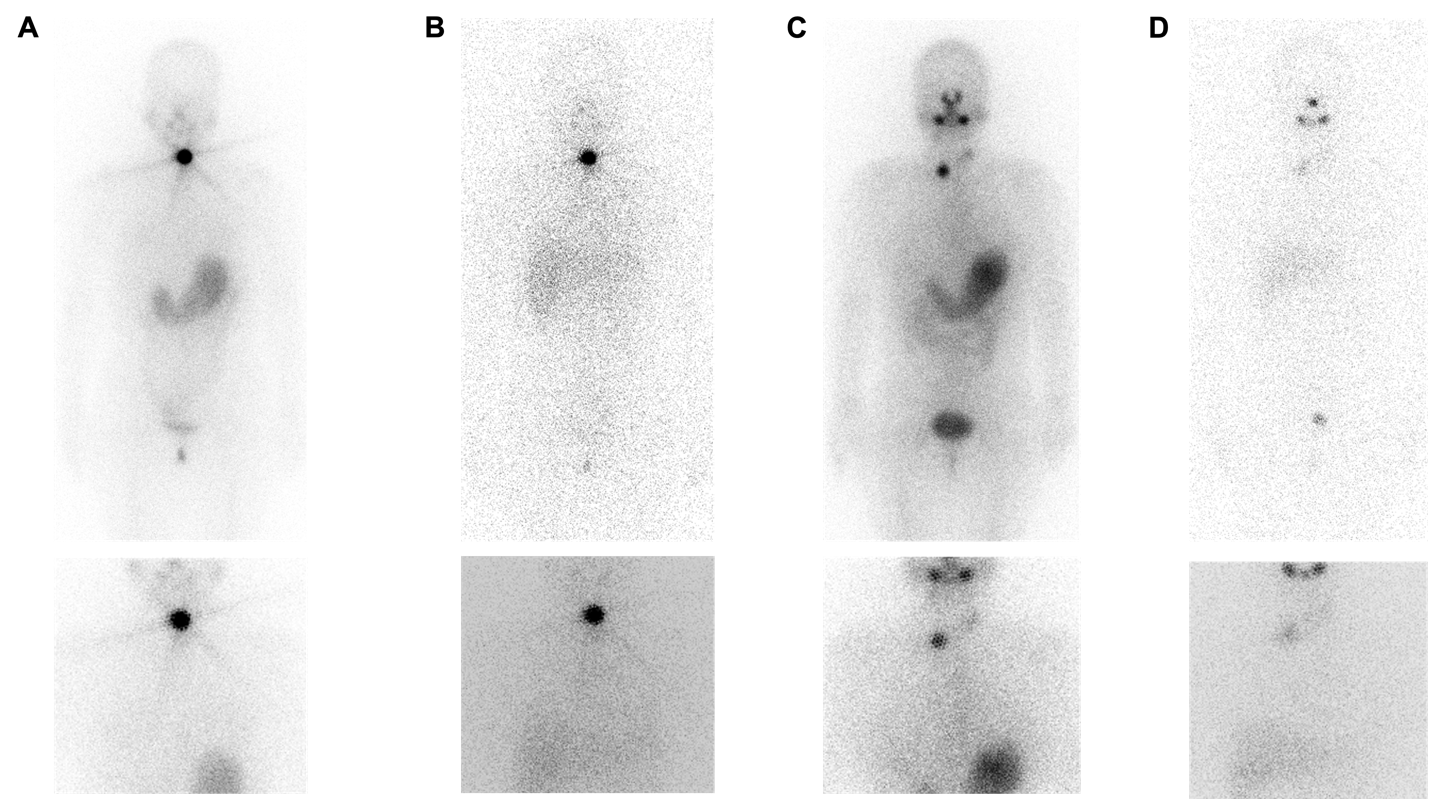


**Supplementary Figure 2.** Calibration plots of the nomogram

**
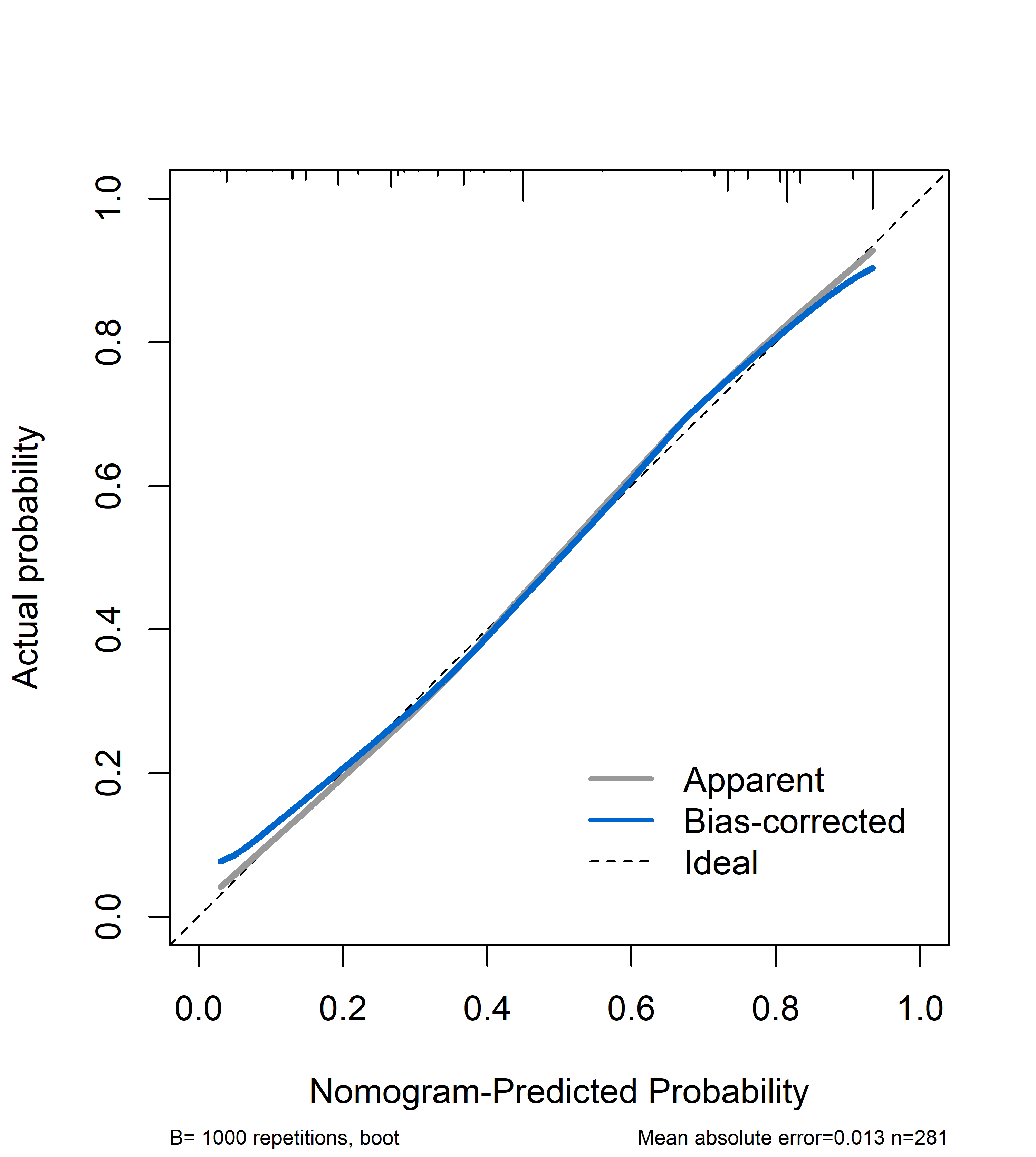
**

**Supplementary Table 1.** Analysis of preoperative serum Tg level associated with pathologic and clinical parameters

| Variable | Univariable | | Multivariable model (r^2^ = 0.25) | |
| --- | --- | --- | --- | --- |
|  | β | P value | β | P value |
| T3N0-1b (vs. T1a-2N1b)  Thyroid glands weight (g) | 10.40  3.68 | 0.523  <0.001 | 2.47 | <0.001 |
| Thyroiditis (vs. absent) | -29.51 | 0.027 | -25.83 | 0.027 |
| Tumor size (cm) | 41.70 | <0.001 | 27.02 | <0.001 |

**Supplementary Table 2.** Correlation between whole body scan neck count and pathologic and clinical parameters

|  | Early WBS neck count^†^ | | | | P value |
| --- | --- | --- | --- | --- | --- |
|  | Q1, n = 71  (656-17,767) | Q2, n= 70  (17,768-49,822) | Q3, n =70  (49,823-114,240) | Q4, n = 70  (114,241-560,227) |  |
| Age (years) | 42 (33.5-55) | 37 (31-44) | 38 (32-50) | 37.5 (30-47) | 0.131 |
| Male:Female | 8:63 | 15:55 | 24:46 | 38:32 | **<0.001** |
| T1a-2N1b:T3N0-1b | 10:61 | 12:58 | 11:59 | 7:63 | 0.650 |
| TSH (mIU/mL) | 51.42 (40.20-64.41) | 48.23 (37.71-65.09) | 51.09 (40.01-76.20) | 52.03 (40.05-65.71) | 0.796 |
| Tg (ng/mL) | 0.2 (0.09-2.5) | 1.3 (0.4-5.2) | 2.7 (1.4-5.3) | 5.9 (3.2-10.6) | **<0.001** |
| TgAb (IU/mL) | 13.5 (9.9-50.4) | 11.4 (9.9-21.3) | 10.0 (9.9-12.7) | 9.9 (9.9-11.9) | **<0.001** |
| Iodine/creatinine ratio | 15.9 (10.8-33.25) | 19.65 (12.4-36.9) | 18.5 (8.5-30.1) | 14.05 (9.1-28.3) | 0.284 |
| Interval between surgery and ablation (months) | 3.0 (2.65-3.55) | 3.1 (2.7-3.6) | 3.2 (2.8-3.6) | 3.2 (2.8-3.8) | 0.701 |
| Thyroiditis, n (%) | 35 (49.3%) | 22 (31.4%) | 11 (15.7%) | 2 (2.9%) | **<0.001** |
|  | Delayed WBS neck count^†^ | | | | P value |
|  | Q1, n = 71  (85-792) | Q2, n= 70  (793-2,378) | Q3, n =70  (2,379-5,974) | Q4, n = 70  (5,975-46,212) |  |
| Age (years) | 41 (33.5-54.5) | 37 (31-46) | 39 (33-52) | 36 (29-48) | 0.115 |
| Male: Female  T1a-2N1b:T3N0-1b | 9:62  10:61 | 16:54  13:57 | 30:40  9:61 | 30:40  8:62 | **<0.001**  0.650 |
| TSH (mIU/mL) | 51.06 (38.69-61.93) | 48.85 (38.71-63.94) | 56.77 (39.95-71.68) | 50.79 (41.69-68.61) | 0.654 |
| Tg (ng/mL) | 0.2 (0.09-2.5) | 1.8 (0.4-5.1) | 2.9 (1.3-6.1) | 4.9 (2.7-10.6) | **<0.001** |
| TgAb (IU/mL) | 13.5 (9.9-82.3) | 11.2 (9.9-21.3) | 11.0 (9.9-14.4) | 9.9 (9.9-11.3) | **<0.001** |
| Iodine/creatinine ratio | 18.4 (11.5-35.3] | 18.5 (11.2-36.6) | 16.9 (8.8-27.1) | 17.8 (11.0-31.3) | 0.650 |
| Interval between surgery and ablation (months) | 3.0 (2.7-3.6) | 3.2 (2.7-3.5) | 3.1 (2.7-3.4) | 3.3 (2.8-3.8) | 0.446 |
| Thyroiditis, n (%) | 37 (52.1%) | 19 (27.1%) | 13 (18.6%) | 1 (1.4%) | **<0.001** |

Continuous values are presented as median (interquartile range).

^†^Scan speed adjusted neck count = neck counts/scan speed (mm/sec)

WBS, whole body scan; Q, quartile; TSH, thyroglobulin stimulating hormone; Tg, thyroglobulin; TgAb, thyroglobulin antibody
